# Impact of the Therapy Capability Framework on the Provision of Physical Health Care in a Large Publicly Funded Mental Health Service

**DOI:** 10.1007/s10597-025-01497-2

**Published:** 2025-08-01

**Authors:** Geoffrey Lau, Justin Chapman, Sally Bennett, Pamela Meredith, Jeanette Sewell, Donni Johnston, Cassandra Butler, Andrea Parker, Marianne Wyder

**Affiliations:** 1 Metro South Health, Woolloongabba, Australia; 2 University of Queensland, Brisbane, Australia; 3 Griffith University, Brisbane, Australia; 4 University of Queensland, Brisbane, Australia; 5 University of the Sunshine Coast, Sunshine Coast, Australia

**Keywords:** Mental health, Therapy capability, Physical health

## Abstract

People with mental illness have poor physical health outcomes. While clinical staff recognise the value of physical health in mental healthcare, they report low confidence and a lack of resources and training. As a result, physical health needs are often unaddressed in routine care. A physical healthcare therapies capability framework (PHC TCF) was developed to support staff in developing capability in addressing the physical health needs of consumers of a large public mental health service. The aims of the current study were to evaluate: (i) staff capability in physical healthcare and the association with provision of service; (ii) changes in capability and service provision. Self-rated PHC TCF level and Provision of Service (POS) statistics for physical health assessment and intervention over 12-months were analysed. The nursing profession was associated with higher PHC TCF level than Allied Health (*p* <.01). While there was no significant change in PHC TCF levels, POS significantly increased over 12 months (*z* = -2.69, *p* =.007). This study demonstrated that a PHC TCF may be a useful tool to reinforce and implement physical health interventions in public mental health services.

## Introduction

In 2020, the total cost of mental illness was between $200 and $220 billion per year which was just over one-tenth of Australia’s entire economic production the previous year (Whiteford, 2021). Mental illness and substance use disorders continue to be a leading contributor of non-fatal burden in Australia, accounting for 24.3% of total years lived with disability, and 14.6% of total disability-adjusted life years for the country’s population (Ciobanu et al., 2018). The largest economised impact is in the form of diminished health and reduced life expectancy. People with mental illness have a shorter life expectancy of 10–20 years compared with the general population, and over three-quarters of this health disparity is caused by preventable physical conditions such as cardiovascular disease (Lawrence et al., 2013). Despite numerous decades of reform agendas at national, state, and territory levels, there has been limited documented evidence that this alarming trend is being overturned (Ciobanu et al., 2018; Mental Health Australia, 2018) with consequential negative impacts including loss of life and reduced productivity (Mindgardens Neuroscience Network, 2019). To assist with delivering better outcomes under the mental health reform agendas, Australian federal and State government reports have urged mental health services to utilise untapped skills amongst the existing professional workforce (Mental Health Workforce Advisory Committee, ( 2011)).

To address the comorbid physical health risks faced by people with mental illnesses in Australia, mental health consumer and service advocates have highlighted the urgent need for specialist mental health services to develop workforce capabilities to prevent and respond to physical health complexities (Cleary et al., 2020). Allied health practitioners, medical officers, mental health nurses, and peer workers make up the key professions working in specialist mental health teams and environments across Australia (Mental Health Workforce Advisory Committee, 2011). Each professional group has profession-specific standards and competencies that encompass biological and physical aspects of an individual’s health determinants (Health, 2013). Despite these expectations, there have been disparities between the perceived roles and responsibilities of the mental health workforce, their confidence, and capabilities when addressing the physical health needs of mental health consumers (Clancy et al., 2019; Howard & Gamble, 2011).

While most staff recognise the value of addressing physical health in mental health care; many clinicians report low confidence in addressing the physical health of consumers in their practice, and a lack of resources or training available to support them (Chapman et al., 2019). As a result, physical health needs are often unaddressed in routine care. Previous studies have suggested the need for more specifically defined duties, targeted training, and supervision to help the mental health workforce to overcome multifactorial barriers that have inhibited them in providing physical health care as an integral part of psychological treatment (Howard & Gamble, 2011; Moyo et al., 2022). Competency refers to a practitioner’s technical ability to undertake a defined set of activities. Capability extends beyond technical skills and strengthens components of adaptability, self-efficacy, and enhanced workforce capacity (Murfet et al., 2022). Whilst a competency framework articulates minimum standards of competence, a capability framework can clarify a pathway for individuals and organisations to adapt and continuously improve practice standards (Network of Alcohol and other Drugs Agencies, 2020). For example, in 2013, the New South Wales (NSW) Public Service Commission published a workforce capability framework for the entire public service sector which was revised in 2020. This resource was designed to provide a large and diverse public service workforce a common language for core capabilities, regardless of level, roles, and occupational backgrounds. Resources, such as this, were not intended to replace specialty competency practice frameworks, but rather complement them (Network of Alcohol and other Drugs Agencies, 2020).

Therapies Capability Frameworks (TCF) may be useful for supporting staff in addressing the shortfall of evidence-based therapy provision by publicly funded mental health services(Lau et al., 2017). The TCF was designed to influence leadership culture; in particular, redefining roles for case managers, supervisors, and managers in planning, implementing, and evaluating evidence-informed psychosocial therapies. In practical terms, this model will help further embed the TCF as an innovation that can improve consumer outcomes by: (a) identifying, supporting, and recognising therapy leaders; (b) investing in clinical workforce skills across all professional groups; and (c) maximising and encouraging interprofessional training (Productivity Commission, 2020; Royal College of Psychiatrists & Royal College of General Practitioners, ( 2008)). A TCF is a matrix that features descriptions of capability “domains” against stratified “levels” of capability. A TCF is used as a reflective tool by the individual practitioner and their supervisor to help direct further capability development, as well as strategically map broader workforce capabilities to identify development needs across an entire organisation (Lau et al., 2017). Within the context of this study, the TCF approach supports clinicians to integrate physical health assessment and intervention in a variety of ways.

### Physical Health Care Therapy Capability Framework to Increase Workforce Capability

In 2021 a physical health care (PHC) TCF was developed and rolled out to enhance the evidence-informed provision of physical health care by all staff, with specific attention to enhancing the role and scope of community-based clinicians in a large public mental health service. The PHC TCF was designed to improve clinical capability of the mental health workforce by outlining a pathway for practitioner knowledge and skills to support physical health and prevent further physical diseases as a core component of service provision. The PHC TCF was co-developed by a Physical Health Working Group which consisted of professionals from dietetics, nutrition, exercise physiology, leisure therapy, occupational therapy, nursing, and the lived experience workforce. Each member of the Physical Health Working Group developed components of the capability framework and accompanying resources based on their professional background and areas of expertise. The PHC TCF *capability domains* include (a) Knowledge and Practice Skills, (b) Autonomy and Supervision, and roles in (c) Research and Evidence-based Practice. The *capability levels* of the PHC TCF range from core capabilities expected of all clinical and peer workforce to most advanced physical health capabilities required for PHC leaders and supervisors. The TCF capability levels from lowest to highest capabilities are Foundation, Practice-informed, Therapist, and Advanced Therapist Levels. A summary of the physical health capability domains across the four capability levels of the PHC TCF is provided in Table 1.

**Table 1:** Summary of capability domains in the PHC TCF

	PHC Capability Levels			
Capability Domains	Foundation Practitioner	Practice-Informed Practitioner	Therapist	Advanced Therapist
Knowledge and Practice Skills	Completes PHC online foundation training module to maintain knowledge and skills as necessary. Coordinates physical health assessment, lifestyle interventions, and person-centred collaborative care.	Employs knowledge and skills in PHC to assess health determinants, conduct individualised needs assessments, health education, and develops recovery-oriented PHC plans with consumers.	Facilitates PHC training for staff, ensures physical health needs of consumers are discussed at MDT case review, and proactive in PHC clinics to maintain quality of care and efficiency.	Leads PHC clinics to ensure fidelity and accountability (e.g., consumer feedback, outcome data, and promotion).
Autonomy and Supervision	Independently employs PHC knowledge and skills within scope and seeks guidance when necessary.	Receives individual or group mentorship from Therapists in PHC professional practice.	Provides mentorship to Practice-Informed staff and receives supervision from Advanced Therapists.	Supervises and develops Practice-Informed staff and Practitioners in PHC. Participates in Advanced Therapist peer mentoring and supervision with other.
Research and EBP	Supports service-based research opportunities and quality improvement initiatives.	Follows organisational guidelines to address each consumer’s determinants of health, supports consumers’ research participation, and participates in PHC quality improvement initiatives.	Participates in local EBP working group, reviews contemporary research and evaluation data (including consumer feedback). Updates guidelines for best practice and assists service-based research and evaluation of usual care.	Chairs EBP working group, coordinates submissions to Quality and Safety Unit to improve usual care practices, and updates guidelines and EBP intervention content. Formulates new research questions and takes a leading role in service evaluation.

*EBP *evidence-based practice; *PHC* physical health care; *MDT* multidisciplinary team

## Method

### Aims

The primary aim of this study was to evaluate changes in staff capability in physical health care and the association with provision of service. A secondary aim was to assess changes in capability and provision of service over time.

### Design

This study is a retrospective evaluation of routine workforce monitoring and provision of service statistics over the period from January to December in 2021; routine care data were de-identified for retrospective analysis. Ethical approvals were obtained from ethics committees of both the Metro South Health and Hospital Service (64065) and The University of Queensland (2020002108).

#### Data included

*Provision of Service (POS)* related to the *number* and *duration* of physical health assessments and interventions calculated as a proportion of total POS for each clinician and summarised as monthly and quarterly averages (Q1 to Q4). Physical health interventions may be education about physical health, support in accessing physical healthcare, or physical activity or nutrition support. Staff who were in direct service provision roles were included in analysis; those in predominantly management or administrative roles were excluded from analysis because the PHC TCF and associated quality improvement activities were designed to impact clinical practice. Only clinicians recorded across the four quarters of 2021 were included to eliminate POS variations over time due to personnel changes.

*Therapy Capability Mapping* Therapy capability mapping was undertaken for professional cohorts (nursing and allied health) between March and April in 2021 (T1) and repeated after six months between September and October in the same year (T2). Only nursing and Allied Health professions were targeted because of their scope of practice in clinical case management within mental health service settings. PHC TCF capability levels (Foundation, Practice-Informed, Therapist, or Advanced Therapist) were self-reported by staff during routine supervision with team leaders, and de-identified for analysis by the researchers. Clinicians’ perceived physical health capability levels were dichotomised into lower level ‘Foundation/Practice-informed’ or higher level ‘Therapist/Advanced Therapist’, because the PHC TCF was designed to have the greatest jump between Practitioner and Therapist levels. This purposeful categorisation distinguishes the functional difference between practitioners and therapists (Lau et al., 2017). Other demographic data, such as gender, was not collated due to the small numbers of each profession per team and the potential for identification.

### Service Context

The study was conducted in a large publicly funded mental health service in Queensland, Australia. The community mental health teams provide care to approximately 2500 adults with mental illness, and comprise over 100 clinicians working in multidisciplinary teams.

### Implementation Strategies

The implementation of the PHC TCF was supplemented by an online learning module and practical resources to support staff access to evidence-informed learning material for developing foundation and practice-informed skills aligned with physical health care capabilities. The online training covered a variety of topics, including evidence about the health gap experienced by people with mental illness, screening for physical health risk factors and diseases, and brief interventions for healthy behaviour change or specialist referral.

### Data Analysis

#### Statistical Analysis

Visual inspection of histograms indicated that the data were non-normally distributed, which was confirmed with Kolmogorov-Smirnov and Shapiro-Wilk normality tests. To determine if any changes occurred to the proportions of physical health POS over time, non-parametric Friedman’s tests and Wilcoxon Signed Rank post-tests (using an adjusted Bonferroni alpha value to avoid type 1 errors) were undertaken for frequency (POS counts) and duration (total minutes) for each of the assessment and intervention categories across Q1 to Q4.

A Chi-square test for independence was used to determine if any significant associations between the frequencies of dichotomous physical health care capability levels and professions were present. To determine if the dichotomous PHC TCF levels changed over time, a McNemar’s test was undertaken between T1 and T2.

Utilising the available data, Mann-Whitney U tests were used to determine if the distribution of physical health POS was different across dichotomous physical health care capability levels. As PHC TCF levels for pre (T1) and post (T2) commenced at different times across geographic areas, media physical health POS statistics from the coinciding 2021 quarter were used for this analysis. For example, if a level 1 PHC TCF capability level was captured in May, the POS data for that variable was taken from Q2.

## Results

### Clinician Characteristics

A total of 104 clinicians were included for assessing physical health POS. Clinicians were tertiary qualified with varying degrees of clinical experience ranging from new graduates to > 20 years working in mental health services. Of the 104 clinicians, 95 (91%) had a recorded PHC TCF level (pre or post) with nine (9%) staff missing PHC TCF data. Professional backgrounds of the participants included nursing (*n* = 46) and allied health staff (*n* = 49), including occupational therapists (*n* = 16), psychologists (*n* = 12), and social workers (*n* = 21). There were different numbers of clinicians for PHC TCF levels captured at T1 (*n* = 84, 81%) and T2 (*n* = 56, 54%); only 49 participants (47%) had PHC TCF levels recorded at both timepoints.

### Capability Levels

**Fig. 1:**
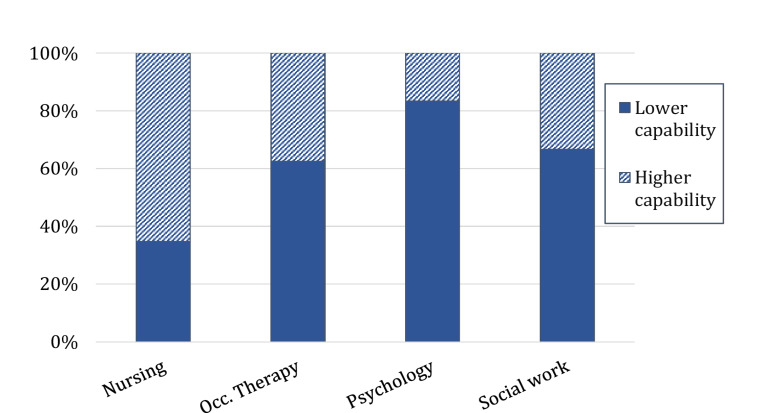
Percentage of dichotomous TCF levels for professions. *Note.* Capability level was dichotomised as “lower” (Foundation or Practice-Informed) or “higher” (Therapist or Advanced Therapist) for nursing (*n*=46), occupational therapy (Occ. Therapy; *n*=16), psychology (*n*=12), and social work (*n*=21). TCF = Therapy Capability Framework

Dichotomised capability levels self-reported as proportions of ‘high capability’ and ‘lower capability’ for each profession are compared in Fig. 1. Overall, the percentages of clinicians in each of the dichotomous levels were similar (53% in ‘lower capability’; 47% in ‘higher capability’). Nursing staff had higher capability levels reported when compared to other Allied Health professionals. (*X*^*2*^ (3, *n* = 95) = 12.70, *p* <.01, *phi* = 0.37).

### Change in Capability Levels

Of the matched participants, only five (10.2%) reported an increase in PHC TCF levels from dichotomous level 1 (Foundation/Practice-Informed) to level 2 (Therapist/Advanced Therapist) over the six-month period. A McNemar’s test indicated that this change in PHC TCF dichotomous levels was nearing statistical significance (*p* =.06).

### Change in POS

The proportions of physical health POS count and durations for each quarter over 12 months (Q1, Q2, Q3, and Q4) are shown in Table 2. The proportion of *total physical health POS* increased between Q1 and Q4, *z* = −2.71, *p* =.007, and the proportion of physical health *interventions* between Q1 and Q4 also increased, *z* = −2.69, *p* =.007. The effect size for both of these increases was *r* =.19, which may represent a modest clinical impact. There were no significant changes in the proportions of physical health assessment provided or duration of overall physical health POS, physical health assessments, or physical health interventions.

**Table 2:** Provision of service statistics for physical health care for 12 months (*n* = 104)

	Service provision for each quarter Median (25th − 75th percentile)				Test statistic ^a^	
	Q1 (Jan–Mar)	Q2 (Apr–Jun)	Q3 (Jul–Sep)	Q4 (Oct–Dec)		
Count (% of total)						
PHC	2.08 (0.69–5.07)	2.37 (0.83–5.71)	2.32 (0.66–5.98)	3.12 (1.08–6.29)	χ^2^(3) = 13.98	*p* =.003*
Assessment	0.41 (0.12–1.77)	0.69 (0.18–2.20)	0.73 (0.18–2.12)	0.89 (0.33–2.42)	χ^2^(3) = 6.55	*p* =.088
Intervention	0.67 (0.12–2.41)	1.09 (0.18–3.71)	1.08 (0.15–3.92)	1.82 (0.26–4.09)	χ^2^(3) = 9.84	*p* =.020*
Duration (% of total)						
PHC	1.96 (0.56–4.45)	2.06 (0.57–8.37)	2.12 (0.57–7.46)	2.78 (0.72–5.86)	χ^2^(3) = 4.09	*p* =.252
Assessment	0.40 (0.06–1.46)	0.50 (0.10–1.08)	0.49 (0.12–1.60)	0.51 (0.17–1.40)	χ^2^(3) = 1.24	*p* =.744
Intervention	0.73 (0.08–2.58)	1.06 (0.22–5.77)	1.17 (0.11–25.0)	1.69 (0.31–4.39)	χ^2^(3) = 4.09	*p* =.252

Note. Provision of service (POS) statistics calculated as a proportion of the total POS for each case manager, as a *count* (number of physical health POS/total number of POS) or *duration* (sum of all physical health POS duration/sum of all POS duration). PHC = physical health care (sum of assessment and intervention POS); Q1–Q4 = Quarter 1 to Quarter 4

^a^ Friedman’s two-way analysis of variance was used to test for trends across the four quarters (Q1–Q4)

* Significance level was Bonferroni corrected: *p* <.05

### Use of Learning Resources

Seven clinicians from adult community mental health teams completed the online physical health care learning module in 2021. This number represents 4.8% of the adult community-based clinicians working at MSAMHS during the study.

## Discussion

This study reported on the results of the implementation of a workforce capability framework, specifically designed for physical health care, commenced in March 2021 across a large public adult community mental health service. The findings indicated that the proportion of clinical services provided by clinicians that were classified as physical health care, increased during the implementation of a physical health workforce capability framework. More physical health interventions per occasions of service were provided in the last quarter of 2021 compared to the first quarter of that same year (*r* =.19).

This change is also reflected in the reports provided by a statewide leadership group called the Mental Health Clinical Collaborative (MHCC). The MHCC routinely distributes monthly data reports for proportions of physical health intervention to all Hospital and Health Services for benchmarking purposes. Throughout the course of this study, these monthly reports also indicated a greater increase in physical health intervention provided by community-based teams when compared to other mental health services that were not in scope for this research. At the completion of this study, two of the three geographic services featured in the top three of 19 services for physical health intervention POS, which was a vast improvement from previous years. This development is particularly notable as the need for more physical health intervention, not just assessment, is the focus for better recovery-oriented care for consumers (Lambert et al., 2017; Productivity Commission, 2020; Queensland Health, 2016).

Regarding the comparison of PHC TCF levels between professional groups, physical health capabilities reported by nurses were significantly higher than allied health professionals. Nurses have been described as critical to the mental health workforce reform agenda in Australia, and possess the necessary expertise to lead, and manage, the physical health needs of consumers during care coordination compared to their interprofessional colleagues (Productivity Commission, 2020). Nurses have also advocated that raising organisational awareness of nurses’ professional physical health expertise can create a better physical health-promoting environment for consumers, as well as nurses within the mental health system (Lundström et al., 2020). The large presence nurses in the service with a self-reported Therapist or Advanced Therapist level of physical health care capability provides an opportunity for sustainable improvement within multi-disciplinary teams if physical health leadership is acknowledged as core professional nursing practice. The presence of nurses working to their full scope of physical health capability could reduce demands without lowering expectations for entire multidisciplinary teams by contributing to broader system-oriented collaborative care partnerships with local GPs and private physical health specialists (Mental Health Australia, 2018); therefore, nurses working to their full scope of physical health capability in mental health settings can play an important role in mentoring their allied health colleagues (Kinnair et al., 2014).

Physical healthcare capability did not change significantly over the evaluation period. This may be because the PHC TCF was insufficiently sensitive to detect changes in capability, particularly given that capability levels are self-reported to team leaders which may be influenced by social desirability bias. Further, only 49 (47%) participants had capability levels reported at both timepoints, which may have reduced sensitivity to change. To build workforce capability in any area of practice, existing clinical expertise needs to be fostered into leadership within teams. The aspiration that therapy capabilities can be enhanced individually and absorbed via peer support over time is hopeful and necessary. If the results of this study resonate with a general understanding that changes in mental health workforce practice requires long-term investment and phased reform (Productivity Commission, 2020), continually recycling training without cultivating leaders from existing professions may delay or prevent implementation of evidence-informed care. In addition, it is a familiar phenomenon for mental health clinicians, in Australia, to face environmental and service-oriented barriers that impede their capacity to actively engage with quality improvement initiatives (Mental Health Workforce Advisory Committee, ( 2011)).

The TCF can be an important tool to change the workforce delivery of different therapies. In the current study, various other initiatives were introduced alongside the PHC TCF, however the uptake of these were very low. Very few clinicians (4.8%) completed the online physical health learning module. Furthermore, workforce interventions, such as POS data reporting audit and feedback, had already been routine practice in previous years. This suggests that simply engaging in the process of using the PHC TCF may have acted as a reminder or reinforcement of the importance of physical health interventions.

### Limitations

The study design did not include a control group; therefore, the influence of the PHC THC on physical health POS is unable to be determined. Unmeasured changes to clinical guidelines, staffing composition, or workforce values and priorities independent of PHC TCF implementation may be responsible for the observed increase in physical health POS. Only clinicians in direct service provision roles were included in analysis (*n* = 104; 72% of all clinicians in community mental health teams), so practice changes for clinicians in strategic or administrative positions are unknown. Further, the proportion of staff who were full-time or part-time, or how this changed over the study period is unknown, which may influenced clinical practice changes. This study was conducted in a publicly funded mental health service in Australia, so the findings may not be generalisable to other service contexts.

Workforce challenges from local clinical contingencies stemming from the COVID-19 pandemic, and organisational barriers and disruptions common to mental health clinicians (Griffiths et al., 2015; McWilliam et al., 2009) were prominent throughout the course of this study. At the time of this study, there were no formal rotations of staff across services or professional streams; however, some teams continued to experience considerable turn-over as high as 30% during 2021. In addition to service restructures and staffing inconsistencies, mandated regulations related to COVID-19 resulted in modifications to clinical service provision. For example, some community-based teams were restricted to telehealth (telephone or online services) whilst other more acute care teams were able to continue in-person contact. The mandated restrictions occurred throughout 2021 and were especially prominent in the last quarter (Q4) as the service prepared for state border re-openings and peak community transmission of COVID-19. Anecdotally, local impacts for the service included more appointment cancellations, less availability of staff, and reduced POS during government enforced community and hospital lockdowns. Despite this, as previously highlighted, the proportions of physical health POS reported by adult services increased.

## Conclusion

Learning and capability strategies for evidence-informed care require targeted and prescriptive communication towards treatment for clinical improvement (Productivity Commission, 2020; Royal College of Psychiatrists & Royal College of General Practitioners, ( 2008)). Significant improvements in the proportion of physical health interventions provided for consumers by public mental health clinicians were detected during the implementation of a bespoke physical health workforce capability framework. The application of physical healthcare capability concepts to assist leaders in promoting evidence-informed care amongst their teams seemed to foster beneficial outcomes in the face of common, and unique challenges experienced by the mental health workforce. While the degree of improvement outcomes attributable to the PHC TCF is unknown, services could promote these outcomes by embedded capability metrics into supervision and training, and encouraging leadership development for clinicians identified as having, or developing, capability in a particular area of practice. More rigorous investigations of targeted workforce capability development strategies to improve physical healthcare in mental health settings are needed to determine the value of this approach for services and consumers. Regardless of this uncertainty, these results present as opportunities to highlight achievements in physical healthcare by clinicians. The urgent improvements needed for mental health service provision may be further delayed, or not realised, if the core practices of professions within the existing mental health workforce are not considered as leadership opportunities for quality improvement.

## Data Availability

Data related to this research is not available for sharing; however, may be available for ethically approved research subject to arrangement.
